# Pst and Tat Systems Are Essential for *Ehrlichia chaffeensis* Intracellular Survival and Regulated by CtrA

**DOI:** 10.3390/microorganisms14081664

**Published:** 2026-07-30

**Authors:** Yuhong Zhou, Mengyao Wang, Yixian Qiu, Shanhua Qin, Mengxiao Li, Zhouyi Chai, Ziyue Qi, Yuqing Sun, Zhihui Cheng

**Affiliations:** 1 The Key Laboratory of Molecular Microbiology and Technology, Ministry of Education, Nankai University, Tianjin 300071, China; 2120231471@mail.nankai.edu.cn (Y.Z.); 2120231469@mail.nankai.edu.cn (M.W.); 2120241537@mail.nankai.edu.cn (Y.Q.); qinshanhua2000@163.com (S.Q.); limengxiao1221@163.com (M.L.); chaizhouyi@126.com (Z.C.); 2110474@mail.nankai.edu.cn (Z.Q.); syq200243@163.com (Y.S.); 2 Department of Microbiology, College of Life Sciences, Nankai University, Tianjin 300071, China

**Keywords:** *Ehrlichia chaffeensis*, human monocytic ehrlichiosis, CtrA, Pst, Tat

## Abstract

*Ehrlichia chaffeensis* is an obligate intracellular Gram-negative bacterium that infects human monocytes and macrophages, causing human monocytic ehrlichiosis (HME). HME is an emerging and highly threatening tick-borne infectious disease. CtrA functions as the response regulator of the CckA/CtrA two-component regulatory system, which exhibits high sequence conservation among α-proteobacteria. As a global transcriptional regulator, CtrA regulates the expression of genes associated with *E. chaffeensis* infection and intracellular survival. Here, we identified consensus CtrA-binding motifs in the promoter regions of genes encoding the phosphate-specific transport (Pst) system and twin-arginine translocation (Tat) system. Using electrophoretic mobility shift assay and reporter assay, our experimental evidence demonstrated that CtrA directly binds to the promoter regions of these target genes and further activates the expression of the corresponding genes. Through complementation experiments, we found that the *E. chaffeensis* Pst and Tat systems are functional and play critical roles in bacterial swimming motility, biofilm formation, and oxidative stress resistance. Using peptide nucleic acid-mediated knockdown, we further confirmed that the Pst and Tat systems are essential for *E. chaffeensis* intracellular survival. Collectively, our data advance the current understanding of the function and regulation of the Pst and Tat systems, which are essential for *E. chaffeensis* intracellular survival, and *E. chaffeensis* pathogenesis and host adaptation.

## 1. Introduction

*Ehrlichia chaffeensis* is an obligate intracellular Gram-negative bacterium that infects human monocytes and macrophages, causing human monocytic ehrlichiosis (HME). As an emerging infectious disease, HME is transmitted by ticks with considerable public health significance. Clinical symptoms of HME include acute fever, headache, chills, myalgia, vomiting, and diarrhea, accompanied by leukopenia, thrombocytopenia, and elevated serum liver transaminase levels. Up to 17% of patients develop life-threatening complications [1]. Severe disease occurs in a subset of patients, particularly among immunocompromised individuals, and fatal outcomes have been reported, which make up 11.6% [2]. Although doxycycline is highly effective when administered early, treatment options remain limited [3]. And no licensed vaccine is currently available. Therefore, investigating the intracellular survival mechanisms of *E. chaffeensis* and identifying effective therapeutic targets have become emerging research priorities.

The CckA/CtrA two-component regulatory system employs CtrA as its response regulator, and this protein is widely conserved among the class α-proteobacteria [4]. Research on *Caulobacter crescentus* has revealed that CtrA regulates the expression of no fewer than 95 genes, which are mainly involved in cell cycle control and polar morphogenesis [5]. Combining chromatin immunoprecipitation with high-throughput deep sequencing (ChIP-seq), our previous study identified a total of 211 target genes whose promoter sequences can be bound by CtrA within the genome of *E. chaffeensis* [6]. It has been demonstrated that in *E. chaffeensis,* CtrA binds to the promoter regions upstream of *bolA*, *surE*, *ompA*, *gshA*, *gshB* and *gst* and activates the expression of these genes [6,7,8]. None of these genes has been previously reported as CtrA downstream genes in *C. crescentus.* These genes are essential for infection and intracellular survival of *E. chaffeensis* [6,7,8]. CtrA in *E. chaffeensis* appears to regulate genes distinct from canonical CtrA downstream genes in *C. crescentus*, suggesting evolutionary repurposing of this regulator during obligate intracellular adaptation.

Among the 211 enriched promoter regions, the promoter regions of *pstB*, *pstC*, *tatA* and *tatC* were respectively enriched 4.03-fold, 1.71-fold, 1.49-fold and 4.92-fold compared with input DNA. These results suggest that CtrA might regulate the expression of the phosphate-specific transport (Pst) system and the twin-arginine translocation (Tat) system in *E. chaffeensis*.

The Pst system is responsible for the uptake of extracellular inorganic phosphate (Pi) into bacteria. In *Escherichia coli*, the Pst system consists of a single operon comprising five genes: *pstS*, *pstA*, *pstB*, *pstC*, and *phoU*, which is regulated by the PhoB/R two-component regulatory system [9,10]. In *Pseudomonas aeruginosa*, the Pst system contains four genes (*pstC*, *pstA*, *pstB*, and *phoU*) within a single operon but lacks *pstS* and its promoter contains a Pho box [11]. The Pst system has been implicated in bacterial pathogenesis. In *Shigella flexneri*, *pst* mutants exhibit reduced plaque size and impaired intracellular proliferation [12]. In *Mycobacterium tuberculosis*, PstB contributes to fluoroquinolone resistance and promotes biofilm formation [13]. Targeting the ATPase activity of PstB has been proposed as a promising strategy against intracellular bacterial pathogens [14]. Notably, the Pst system in *E. chaffeensis* is minimal and contains only *pstA*, *pstB* and *pstC*, lacking *pstS* and *phoU* [15]. These genes are scattered throughout the genome but not in an operon. As an obligate intracellular bacterium that inhabits a Pi-poor host cytoplasmic environment [16], *E. chaffeensis* relies on high-affinity Pi uptake for survival. However, whether its Pst system contributes to infection and intracellular survival as well as its regulatory mechanism remains unclear.

The Tat system is located in the inner membrane and mediates the export of fully folded proteins from the cytoplasm to the periplasm [17]. In *E. coli*, the Tat system includes *tatA*, *tatB*, *tatC*, and *tatE* (a paralog of *tatA*) [18,19]. The core Tat system in *P. aeruginosa* is identical to that in *E. coli*, consisting of the *tatA*, *tatB*, and *tatC* genes in a single operon [20]. It is involved in the secretion of virulence factors, iron acquisition, anaerobic metabolism, and biofilm formation [21]. Previous studies have linked the Tat system to bacterial intracellular survival and pathogenicity. In *Brucella ovis*, *tat* mutants display increased sensitivity to H_2_O_2_, membrane defects, reduced intracellular survival, and attenuated virulence [22]. The genes encoding the Tat system (*tatA*, *tatB*, and *tatC*) disperse throughout all rickettsial genomes [23], indicating that the Tat system constitutes a conserved secretion pathway that may be required for bacterial infection and intracellular survival. In *Anaplasma phagocytophilum*, a high expression of *tatA* supports the adaptation to obligate intracellular parasitism [24]. *E. chaffeensis* possesses a Tat system, with *tatA*, *tatB* and *tatC* dispersed in the genome. The roles of the Tat system in the intracellular survival and pathogenicity of *E. chaffeensis* as well as its regulatory mechanism remain unknown.

In this study, using the electrophoretic mobility shift assay and reporter assay, we demonstrate that CtrA directly binds to the promoter regions of *pstA*, *pstB*, *pstC*, *tatA*, *tatB* and *tatC* and activates their expression. Through complementation experiments, we demonstrate that the *E. chaffeensis* Pst and Tat systems retain conserved biological activity and can rescue the defects of bacterial swimming motility, biofilm formation, and oxidative stress resistance in *P. aeruginosa* mutants. Using peptide nucleic acid (PNA)-mediated knockdown, we further confirmed that the Pst and Tat systems are important for *E. chaffeensis* intracellular survival.

## 2. Materials and Methods

### 2.1. Bacterial Strains and Cell Culture, Plasmids and Primers

The Arkansas strain of *E. chaffeensis* was propagated in the human acute leukemia THP-1 cell line, as previously described [25]. The THP-1 cell line was obtained from Fuheng Biotechnology (Shanghai, China, FH0112). The infected and uninfected cells were maintained in RPMI 1640 medium supplemented with 2 mM L-glutamine and 10% (*v*/*v*) fetal bovine serum (FBS; Every Green, Huzhou, China), under the conditions of 37 °C, 5% CO_2_, and 95% air. The cell culture was routinely examined with a TransDetect qPCR Mycoplasma Detection Kit (TransGen Biotech, Beijing, China) to make sure it was free of *Mycoplasma* contamination. *E. coli* strain DH5α was used for DNA cloning and BL21 (DE3) served as the host strain for protein expression. Both strains were cultured in Luria–Bertani (LB) broth as described previously [8]. Antibiotics including gentamicin (50 μg/mL) (Solarbio, Beijing, China), chloramphenicol (34 μg/mL) (Macklin, Shanghai, China) and ampicillin (100 μg/mL) (Sangon Biotech, Shanghai, China) were added to the culture medium depending on experimental requirements. To study the function of *E. chaffeensis* PstB or TatC, *P. aeruginosa* PA14 strain (wild-type), PA14 strain mutant with Transposon (Tn) insertion in the PA5366 (*pstB*::Tn) or PA5070 (*tatC*::Tn) from the PA14 Non-Redundant Transposon Insertion Mutant Set (PA14NR Set) [26] and complemented strains were cultured in LB broth as described previously [27]. When required, carbenicillin (150 μg/mL) (Shyuanye, Shanghai, China) and gentamicin (50 μg/mL) were supplemented into the medium for selective cultivation.

The bacterial strains, plasmids, PNAs, and ssRNAs used in this study are listed in Supplementary Materials, Table S1. Primers used in this study are listed in Supplementary Materials, Table S2.

### 2.2. Isolation of E. chaffeensis

*E. chaffeensis* was isolated from infected THP-1 cells (90% infection, 2 × 10^7^ cells/mL) as described previously with minor modifications [28]. Infected cells were harvested by centrifugation at 500× *g* for 5 min at room temperature, then resuspended in 5 mL SPK buffer (200 mM sucrose, 50 mM potassium phosphate, pH 7.4) and lysed by passing the suspension 30 times through a 23-gauge needle on ice. To remove cell debris and unbroken cells, the lysate was centrifuged at 1000× *g* for 5 min at 4 °C. The supernatant was further centrifuged at 10,000× *g* for 10 min at 4 °C. The resulting pellet was resuspended in 0.3 M sucrose for PNA transfection.

### 2.3. PNA Transfection

Antisense PNAs targeting *E. chaffeensis ctrA*, *pstB* and *tatC* genes were synthesized by Hangzhou Taihe Biotechnology Co., Ltd. (Hangzhou, China; Supplementary Table S1). These PNAs were used to knock down the expression levels of the corresponding genes as described previously with minor modifications [6]. To confirm the specific interaction between PNAs and their corresponding target sequences, ssRNAs derived from *pstB* and *tatC* were synthesized by GENTLEGEN (Suzhou, China; Supplementary Table S1). A total of 40 pmol of ssRNA was incubated in the presence of 20 pmol gene-targeting PNA or scrambled control PNA (CTL-PNA) in hybridization solution supplemented with 250 mM Tris-HCl (pH 7.2) to reach a final reaction volume of 15 μL. Hybridization was performed on a BIO-GENER GE4852T thermocycler (Hangzhou, China) with the following program: 95 °C for 3 min, then gradual cooling to 35 °C over 60 cycles (20 s per cycle). Subsequently, the prepared hybridization samples were separated via 12% native polyacrylamide gel, followed by GelGreen nucleic acid staining (Biomed, Beijing, China). Bands were imaged with a ChemiDoc XRS+ Molecular Imager (BIO-RAD, Hercules, CA, USA).

To knock down the expression of a specific gene, 3 μg of PNA or CTL-PNA dissolved in nuclease-free water was mixed with 100 μL of host cell-free *E. chaffeensis* resuspended in 0.3 M sucrose and incubated on ice for 15 min. Using a Bio-Rad Gene Pulser Xcell electroporation system (Hercules, CA, USA), electroporation was performed in a 2 mm Bio-Rad cuvette with a 10 ms pulse (2000 V, 10 kV/cm, 25 μF, 400 Ω). Subsequently, the PNA-transfected *E. chaffeensis* was seeded into T25 cell culture flasks to infect 5 × 10^5^ THP-1 cells. The cells were incubated at 37 °C for 2 h, with gentle shaking conducted every 15 min to facilitate the uptake of bacteria by host cells. At 36 h post infection (h p.i.), infected cells were collected and quantitative RT-PCR (qRT-PCR) was used to confirm the gene knockdown efficiency.

### 2.4. Quantitative RT-PCR

Total RNA was extracted from collected samples and reverse-transcribed into complementary DNA (cDNA) as described previously with minor modifications [29]. Total RNA was extracted using a TransZol Up Plus RNA Kit (TransGen Biotech) according to the manufacture’ s protocol and the quality of RNA was assessed using a NanoDrop 2000 Spectrophotometer (Thermo Fisher Scientific, Rockford, IL, USA). The cDNA was synthesized from the total RNA using HiScript III RT SuperMix for qPCR (+gDNA wiper) (Vazyme, Nanjing, China). Quantitative PCR (qPCR) was performed to detect the expression levels of *E. chaffeensis ctrA*, *pstA*, *pstB*, *pstC*, *tatA*, *tatB*, *tatC*, 16S rRNA, and human *GAPDH* genes using gene-specific primers (Supplementary Table S2). The reactions were conducted using PerfectStart Green qPCR SuperMix (TransGen Biotech) on a StepOnePlus Real-Time PCR System (Applied Biosystems, Woburn, MA, USA). The specificities and efficiencies were assessed with the melting curves. The relative expression levels of *ctrA*, *pstA*, *pstB*, *pstC*, *tatA*, *tatB* or *tatC* were normalized against those of *E. chaffeensis* 16S rRNA. The bacterial numbers were calculated as the ratio of *E. chaffeensis* 16S rRNA level to human *GAPDH* mRNA level.

### 2.5. Expression and Purification of Recombinant Proteins

The DNA fragment encoding full-length *E. chaffeensis ctrA* was cloned into the pET-41a(+) vector to construct the plasmid expressing the recombinant *E. chaffeensis* CtrA fused with a GST tag and a His tag (designated as GST-rCtrA) as described previously with minor modifications [8]. This recombinant plasmid or the empty pET-41a(+) vector as a negative control was electrotransformed into *E. coli* BL21 (DE3) competent cells. Once the bacterial culture reached an OD_600_ of 0.4–0.6, the recombinant protein expression in *E. coli* BL21 (DE3) was induced using 0.1 mM isopropyl-thio-β-D-galactoside (IPTG; TaKaRa, Beijing, China) at 37 °C for 4 h. GST-rCtrA and GST proteins were purified from the soluble fraction of *E. coli* lysates via Ni-affinity chromatography, then dialyzed in stocking buffer (10 mM Tris-HCl, pH 7.5, 1 mM dithiothreitol).

### 2.6. Electrophoretic Mobility Shift Assay

The electrophoretic mobility shift assay (EMSA) was performed as described previously with minor modifications [6]. The promoter regions of *E. chaffeensis pstA* (201 bp), *pstB* (300 bp), *pstC* (182 bp), *tatA* (332 bp), *tatB* (183 bp), *tatC* (198 bp), and *pstB* encoding region (negative control) (101 bp) were PCR-amplified using gene-specific primers (Supplementary Table S2). DNA probes of 50 ng were mixed with 2.5 μM purified GST-rCtrA or GST in a 20 μL reaction buffer (10 mM Tris-HCl pH 7.5, 1 mM DTT, 50 mM KCl) and incubated on ice for 30 min. After an 8% native polyacrylamide gel (1 × TBE) was pre-run at 100 V for 1 h, the reaction samples were loaded, and electrophoresis was run at 10 mA on ice for 1.5 h. The gel was stained with 1 × TBE containing 0.1 μL/mL StarStain Red Enhanced Nucleic Acid Dye (GenStar, Beijing, China) at room temperature for 10 min, and bands were imaged with the ChemiDoc XRS+ Molecular Imager (BIO-RAD).

### 2.7. Construction of Enhanced Green Fluorescent Protein Fusions and Reporter Assay

The construction of eGFP fusion plasmids and reporter assay were performed as described previously with minor modifications [6]. The promoter region of *E. chaffeensis pstA*, *pstB*, *pstC*, *tatA*, *tatB*, *tatC* or *p28* (negative control) was inserted upstream of the promoter-less *egfp* gene in the pQE60 vector to generate an eGFP fusion plasmid. *E. coli* BL21 (DE3) harboring pACYCDuet-1-rCtrA or empty pACYCDuet-1 (negative control) was electrotransformed with these plasmids. At an OD_600_ of 0.4–0.6, these *E. coli* strains were induced with 0.05 mM IPTG at 37 °C for 3 h to express target proteins. Equal biomass bacteria were collected, and Western blotting was used to detect the expression levels of eGFP, CtrA and RpoA (internal reference).

### 2.8. Western Blotting

Western blotting analysis was performed as described previously with minor modifications [28]. *E. coli* cells were harvested by centrifugation at 12,000× *g* for 1 min, resuspended in 1 × SDS loading buffer, and boiled for 10 min. Proteins were separated by 12% SDS-polyacrylamide gel electrophoresis (SDS-PAGE) and then transferred onto a PVDF membrane (Millipore, Co., Cork, Ireland). Subsequently, the membrane was probed with mouse monoclonal anti-GFP antibody (1:2000 dilution) (GeneTex Inc., Irvine, CA, USA, GTX628528), mouse monoclonal anti-*E. coli* RNA polymerase α antibody (1:2000 dilution) (BioLegend, San Diego, CA, USA, 663104), or rabbit polyclonal anti-CtrA antiserum (1:1000 dilution) for target protein detection. The membranes were washed four times with PBST (137 mM NaCl, 10 mM Na_2_HPO_4_, 2 mM KH_2_PO_4_, 2.7 mM KCL, 0.1% (*v*/*v*) Tween 20), followed by incubation with HRP-conjugated goat anti-rabbit IgG H&L secondary antibody (1:2000 dilution) (Promega, Madison, WI, USA, W4011) or HRP-conjugated goat anti-mouse IgG H&L secondary antibody (1:2000 dilution) (Promega, W4021), respectively, at room temperature for 1 h. The bands were imaged with the ChemiDoc XRS+ Molecular Imager (BIO-RAD). The expression level of eGFP in each sample was normalized against that of RpoA in the corresponding sample.

### 2.9. Swimming Motility Assay

The swimming motility assay was performed as described previously with minor modifications [8]. Overnight cultured *P. aeruginosa* strains were diluted to an OD_600_ of 0.1. A total of 1 μL of the diluted suspension was spotted onto swimming medium plates (1% (*w*/*v*) tryptone, 0.5% (*w*/*v*) NaCl, and 0.3% (*w*/*v*) agarose), which were then cultured at 30 °C for 16 h. Bacterial colonies were imaged using the ChemiDoc XRS+ Molecular Imager (BIO-RAD) and their diameters were measured.

### 2.10. Biofilm Assay

Biofilm formation was examined as described previously with minor modifications [30]. *P. aeruginosa* strains cultured overnight were resuspended and diluted in LB broth to an OD_600_ of 0.03 and cultured at 37 °C to an OD_600_ of 1.0, then diluted to an OD_600_ of 0.25 in LB broth. A total of 150 μL of the suspension was inoculated into each well of a 96-well plate, followed by incubation at 37 °C for 24 h. Each well was washed three times with 1 × PBS, dried for 20 min, stained with 0.25% (*w*/*v*) crystal violet for 20 min, then washed twice with 1 × PBS. A total of 200 μL of methanol was added to each well for 15 min at room temperature to solubilize crystal violet. Quantification was performed by measuring OD_590_ using a Varioskan Flash microplate reader (Thermo Scientific, Billerica, MA, USA).

### 2.11. H_2_O_2_ Assay

The susceptibility of *P. aeruginosa* to H_2_O_2_ was measured following a previously described protocol with slight adjustments [27]. Overnight *P. aeruginosa* cultures were diluted in LB broth to an OD_600_ of 0.03 and cultured at 37 °C. At an OD_600_ of 1.0, bacteria from 1 mL of culture were collected and washed three times with 1 × PBS, then resuspended in 1 × PBS buffer supplemented with 50 mM H_2_O_2_ at 37 °C for 30 min. Serial dilution combined with colony enumeration was adopted to quantify bacterial survival rates.

### 2.12. Statistical Analysis

All experiments were performed for at least three times of biological replicates. Analyses were performed using GraphPad Prism 9.5.1. Student’s *t*-test (two-tailed) was utilized for the significance of a two-group comparison. One-way analysis of variance (ANOVA) was utilized for the significance across three or more groups. Data are presented as mean ± standard deviation. A value of *p* < 0.05 was considered significant.

## 3. Results

### 3.1. CtrA Is Involved in the Regulation of pstA, pstB, pstC, tatA, tatB and tatC in E. chaffeensis

**Figure 1:**
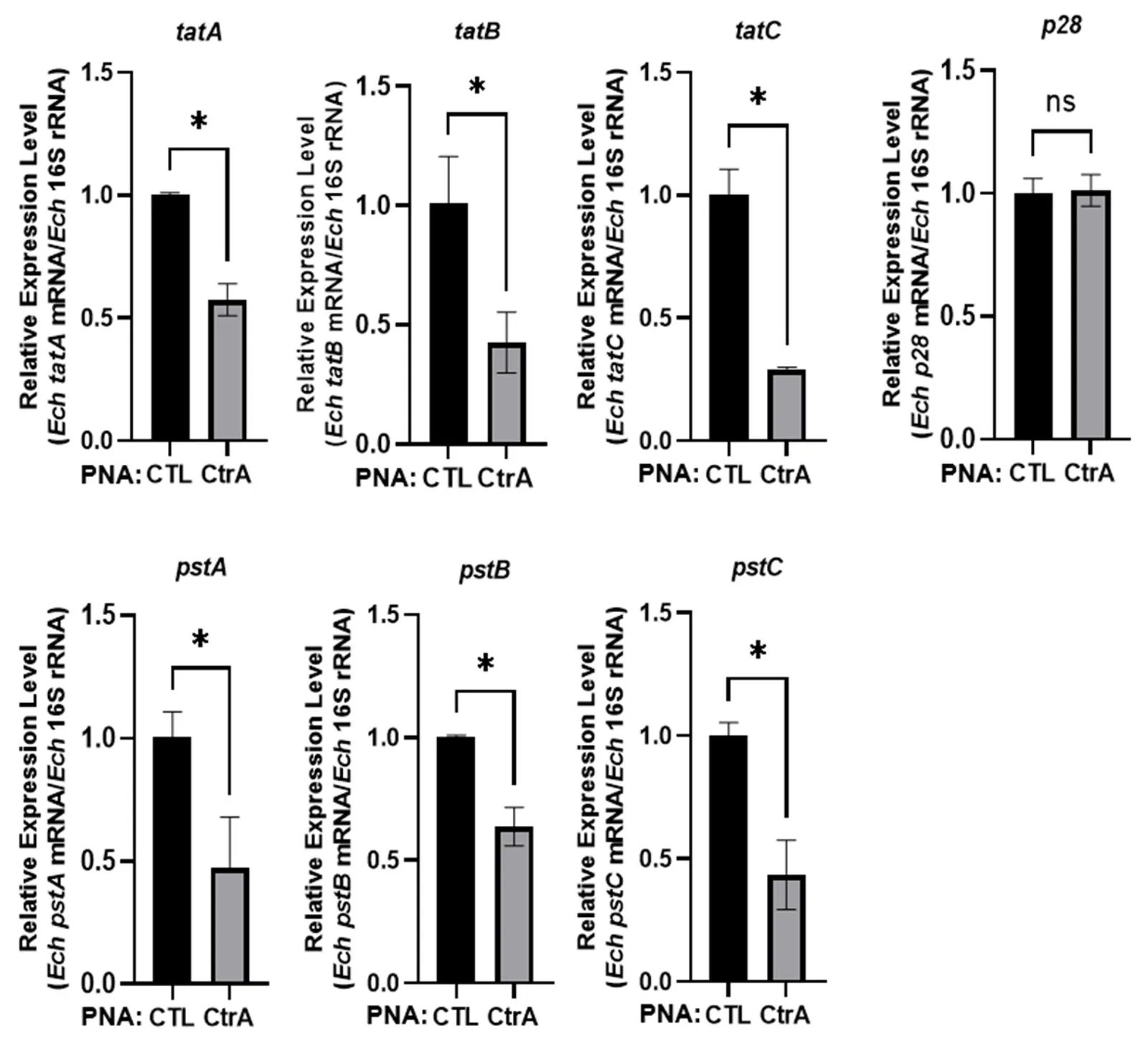
Transfection with CtrA PNA inhibits the expression of *pstA*, *pstB*, *pstC*, *tatA*, *tatB* and *tatC*. THP-1 cells synchronously infected with CTL PNA- or CtrA PNA-transfected *E. chaffeensis*. The mRNA expression levels of *pstA*, *pstB*, *pstC*, *tatA*, *tatB* and *tatC* were determined with qRT-PCR at 36 h p.i. and normalized against the *E. chaffeensis* 16S rRNA levels. Relative values to the amount of CTL PNA-transfected *E. chaffeensis* are shown. All data are presented as mean ± standard deviation (n = 3), with significant difference determined by Student’s *t*-test (* *p* < 0.05). ns, no significant difference.

The promoter regions of *pstB*, *pstC*, *tatA* and *tatC* were enriched by CtrA in ChIP-seq [6], and *pstA* and *tatB* also exist in the genome of *E. chaffeensis.* PstA and PstC form the transmembrane channel and PstB acts as a cytoplasmic ATPase to provide energy for transport [10]. TatB and TatC form the substrate recognition complex that binds the twin-arginine signal peptide, and TatA polymerizes to form the translocation channel for folded or cofactor-containing proteins [19]. It is highly likely that a regulator is required to activate the expression of all genes encoding the Pst or Tat systems at the same time to ensure that the complexes are assembled when they are needed in *E. chaffeensis*. We firstly investigated whether the expression of these genes is regulated by CtrA in *E. chaffeensis*. No *E. chaffeensis* deletion mutant is currently available, because classical bacteriology techniques are not readily applicable for this obligatory intracellular bacterium [31]. PNA is a DNA mimic that has been shown to bind single- and double-stranded RNA and DNA with high affinity and specificity [32], and can be used to knock down *E. chaffeensis* proteins [6,8,33]. Previously, we used PNA to specifically knock down the levels of *ctrA* mRNA and CtrA proteins in *E. chaffeensis* [8]. Here we examined the expression of *pstA*, *pstB*, *pstC*, *tatA*, *tatB* and *tatC* after CtrA knockdown in *E. chaffeensis*. Host cell-free *E. chaffeensis* was transfected with CtrA PNA or a scrambled control PNA (CTL PNA), then used to infect THP-1 cells. At 36 h p.i., when bacterial growth reached the early exponential phase [7], *ctrA* mRNA level and bacterial number were significantly reduced to 48.3% and 16.6%, respectively, with CtrA PNA transfection compared with those with CTL PNA transfection in line with previous results (Supplementary Figure S1) [6,8]. We found that the transfection with CtrA PNA reduced the expression levels of *pstA*, *pstB*, *pstC*, *tatA*, *tatB* and *tatC* to 46.8%, 63.7%, 43.5%, 57.4%, 42.1% and 28.8%, respectively, in *E. chaffeensis* (Figure 1), while the expression level of a non-targeting gene *p28* showed no changes. These results suggest that CtrA may regulate the expression of *pstA*, *pstB*, *pstC*, *tatA*, *tatB* and *tatC* in *E. chaffeensis*.

### 3.2. CtrA Directly Binds to the Promoter Regions of pstA, pstB, pstC, tatA, tatB and tatC

**Figure 2:**
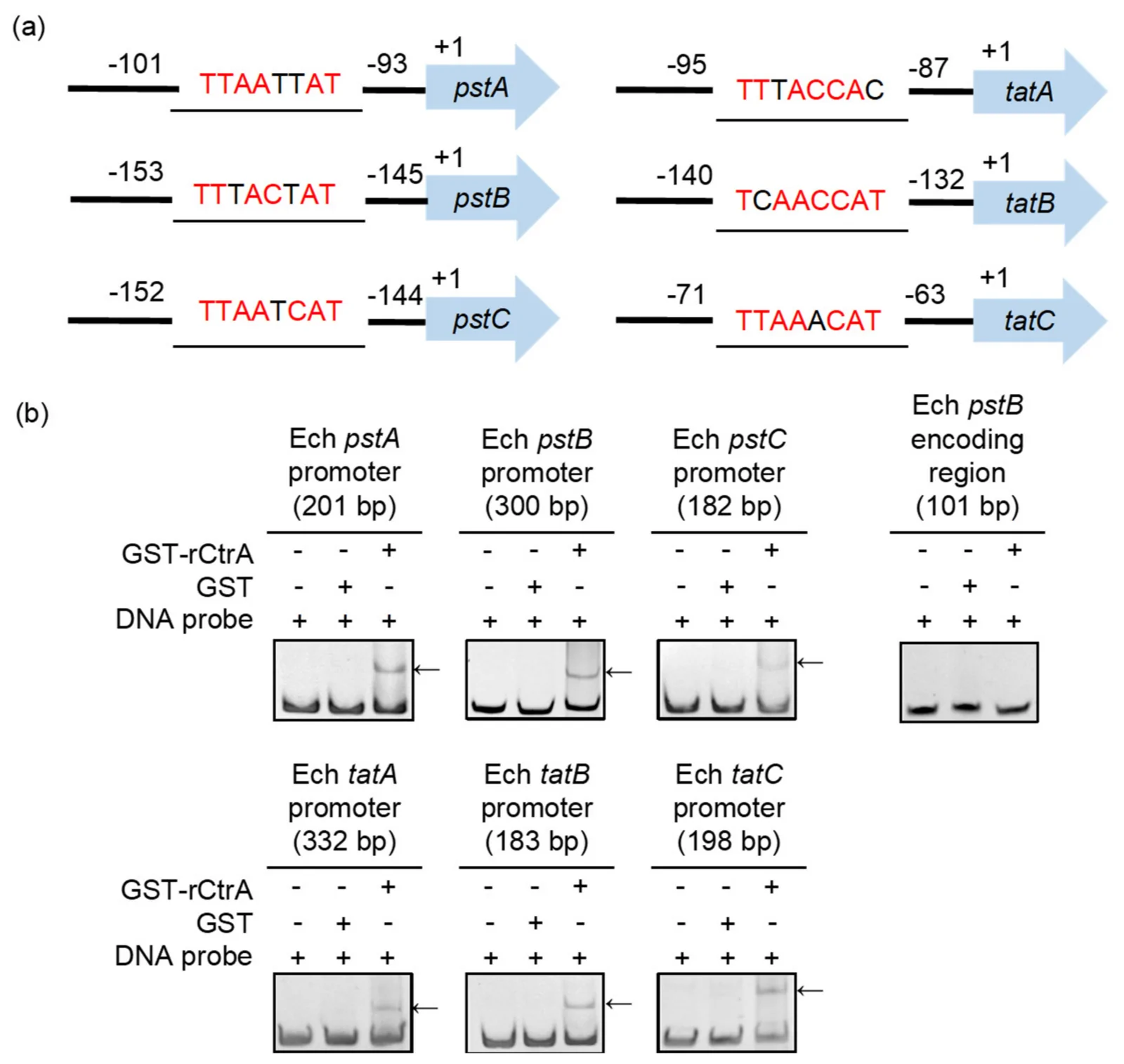
CtrA binds to the promoter regions of *pstA*, *pstB*, *pstC*, *tatA*, *tatB* and *tatC*. (**a**) Consensus CtrA-binding motifs in the promoter regions of *pstA*, *pstB*, *pstC*, *tatA*, *tatB* and *tatC*. Arrows represent the transcriptional direction of these genes. Numbers represent the positions of the consensus CtrA-binding motifs in the promoter regions of these genes calculated from the translational start codons. Black letters represent the mismatched bases. (**b**) DNA probes (50 ng) were incubated alone, with GST (2.5 μM) or GST-rCtrA (2.5 μM) on ice for 30 min. Shifted bands are indicated by arrows. The *pstB* encoding region was used as a negative control. The name and length of each promoter region are indicated above the corresponding panel.

We then screened the promoter regions of *pstA*, *pstB*, *pstC*, *tatA*, *tatB* and *tatC* for the consensus CtrA-binding motifs (9-mer, TTAAN_7_TTAAC; 8-mer, TTAACCAT) and found that each promoter region of these genes contains a consensus CtrA-binding motif (Figure 2a). We next investigated whether CtrA binds to the promoter regions of *pstA*, *pstB*, *pstC*, *tatA*, *tatB* and *tatC* using GST-rCtrA [6]. The purified GST-rCtrA showed a single band on the SDS-PAGE gel (Supplementary Figure S1). The DNA probes derived from the promoter regions of *pstA*, *pstB*, *pstC*, *tatA*, *tatB* and *tatC* were shifted upon the incubation with GST-rCtrA (Figure 2b). The binding specificity was confirmed by the results that no shifted bands were detected when the DNA probes were incubated with purified GST protein or when using a DNA probe derived from the *pstB* encoding region, which does not contain the consensus CtrA 8-mer or 9-mer binding motif allowing 1 or 2 bp mismatch (Figure 2b). These results indicate that CtrA directly binds to the promoter regions of *pstA*, *pstB*, *pstC*, *tatA*, *tatB* and *tatC* in *E. chaffeensis*.

### 3.3. CtrA Activates the Expression of pstA, pstB, pstC, tatA, tatB and tatC

**Figure 3:**
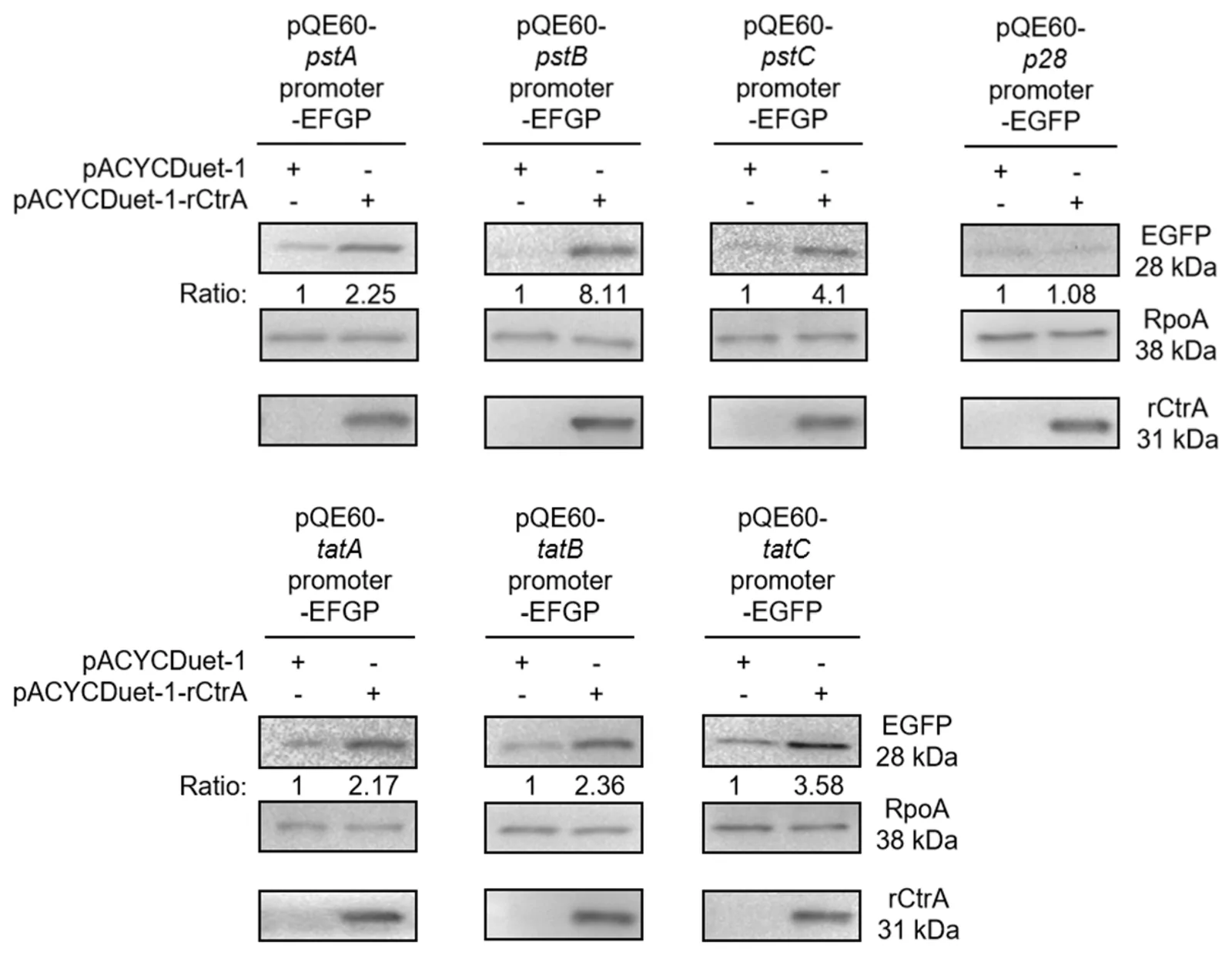
CtrA activates the expression of *pstA*, *pstB*, *pstC*, *tatA*, *tatB* and *tatC*. *E. coli* BL21 (DE3) strains were grown to an OD_600_ of 0.4–0.6, then induced with 0.05 mM IPTG at 37 °C for 3 h to express rCtrA. The levels of EGFP and RpoA were determined by Western blotting. The RpoA levels in each panel show that the two lanes received the same amount of bacteria. The relative intensities of EGFP to those of RpoA were measured by Image J (1.49v, National Institutes of Health, USA). The relative intensity from the strain containing pACYCDuet-1 plasmid in each panel was set as 1.

We then examined whether CtrA activates the expression of *pstA*, *pstB*, *pstC*, *tatA*, *tatB* and *tatC*. Due to the lack of effective genetic manipulation methods in *E. chaffeensis*, we used an *E. coli* reporter assay system [6,8]. The promoter region of *pstA*, *pstB*, *pstC*, *tatA*, *tatB*, *tatC*, or *p28* (no consensus CtrA-binding motif, negative control) was inserted upstream to the promoter-less *egfp* gene in the pQE60 plasmid to generate *pstA* promoter–EGFP, *pstB* promoter–EGFP, *pstC* promoter–EGFP, *tatA* promoter–EGFP, *tatB* promoter–EGFP, *tatC* promoter–EGFP or *p28* promoter–EGFP fusion plasmid, respectively. The *E. coli* BL21 (DE3) strain containing the pACYCDuet-1 vector harboring the *E. chaffeensis ctrA* gene (pACYCDuet-1-rCtrA) or the pACYCDuet-1 vector only (negative control) was respectively transformed with each EGFP fusion plasmid. The rCtrA expression induced with IPTG resulted in a significantly higher expression of EGFP in bacteria harboring *pstA* promoter–EGFP, *pstB* promoter–EGFP, *pstC* promoter–EGFP, *tatA* promoter–EGFP, *tatB* promoter–EGFP, or *tatC* promoter–EGFP compared with the vector control (Figure 3). And the rCtrA expression had no effects on bacteria harboring *p28* promoter–EGFP fusion plasmid (Figure 3). These results indicate that CtrA activates transcription from these promoter regions in a heterologous *E. coli* reporter system.

### 3.4. E. chaffeensis PstB and TatC Are Functional

**Figure 4:**
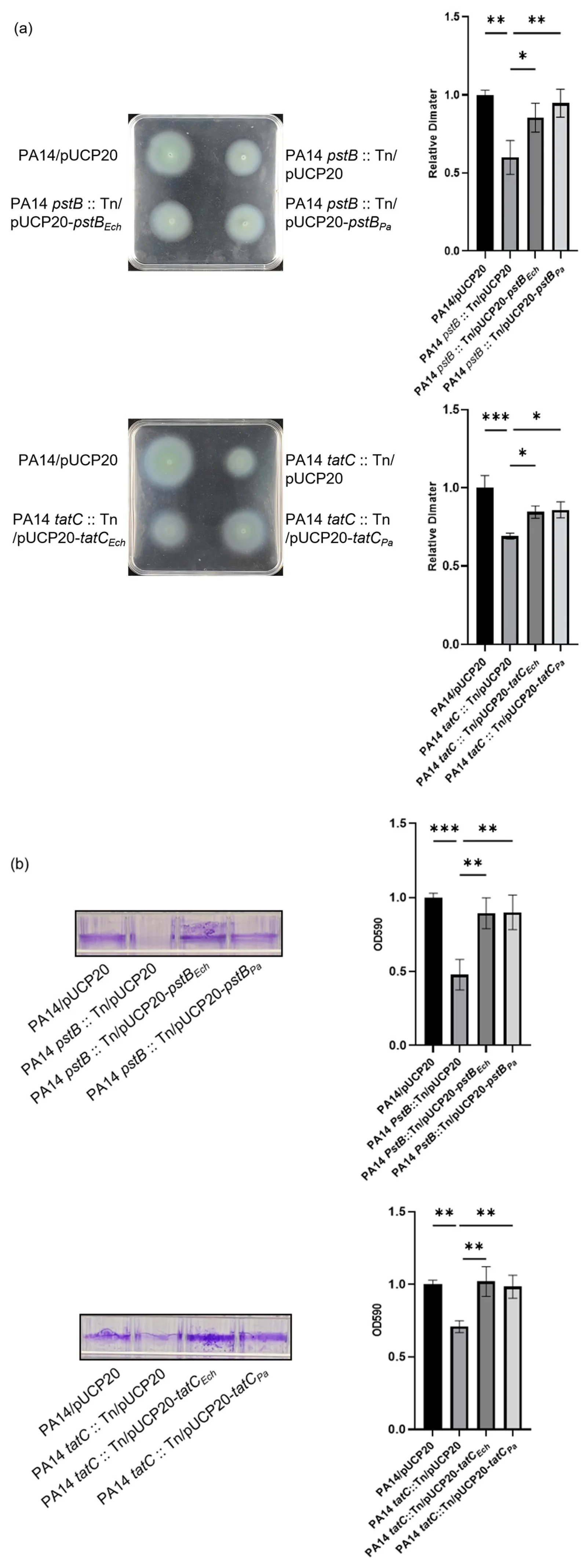

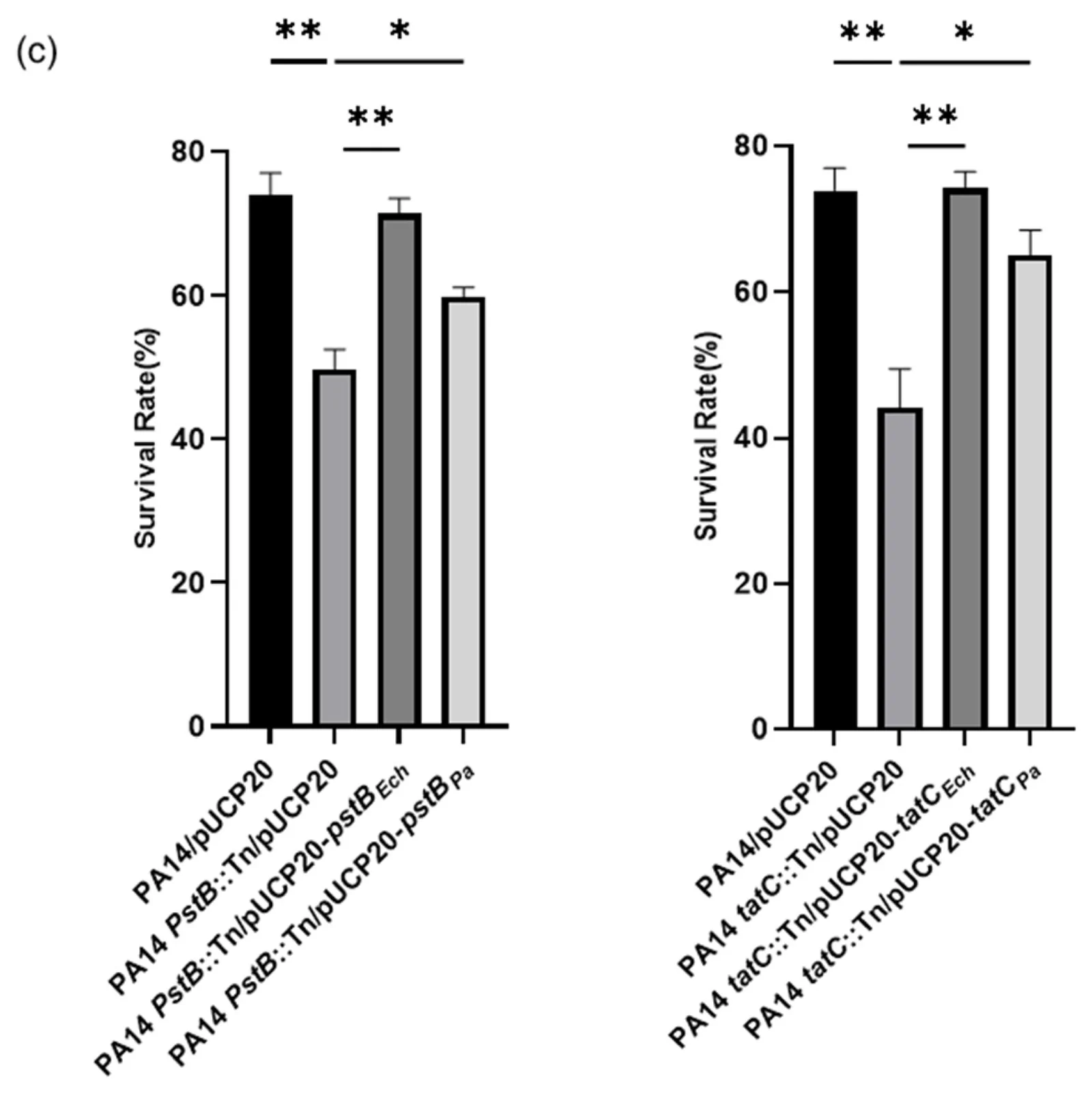
*E. chaffeensis* PstB or TatC restores the swimming motility, biofilm formation and survival under oxidative stress of the *P. aeruginosa pstB*::Tn or the *tatC*::Tn mutant. (**a**) *E. chaffeensis* PstB or TatC restores the swimming motilities of the corresponding *P. aeruginosa* mutant strain. A total of 1 μL aliquot of diluted overnight culture (OD_600_ = 0.1) of each strain was inoculated onto the agar plates and incubated at 30 °C for 16 h (left). Swimming motility was assessed by measuring the diameter of the outermost spreading edge of each colony. Relative values to the diameter of the wild-type PA14 strain are shown (right). (**b**) *E. chaffeensis* PstB or TatC restores the biofilm formation of the corresponding *P. aeruginosa* mutant strain. Overnight cultures of each strain were subcultured to an OD_600_ of 1.0, then diluted to an OD_600_ of 0.25 in LB medium. A total of 150 μL of the diluted cultures was added to 96-well plates and incubated at 37 °C for 24 h. Biofilm formation was assessed by staining with 0.25% (*w*/*v*) crystal violet (left), followed by measurement of the OD_590_ of the methanol eluate (right). Relative values to the measurement of the wild-type PA14 strain are shown. (**c**) *E. chaffeensis* PstB or TatC confers survival ability under oxidative stress to the corresponding *P. aeruginosa* mutant strain. *P. aeruginosa* strains grown to an OD_600_ of 1.0 were treated with H_2_O_2_ at a final concentration of 50 mM at 37 °C for 30 min. The survival rate is shown as the ratio of the colony number of each strain treated with H_2_O_2_ to that of the untreated group. All data are presented as mean ± standard deviation (n = 3), with significant difference determined by one-way ANOVA (* *p* < 0.05, ** *p* < 0.01, *** *p* < 0.001). Representative images from a single experiment are shown.

We then investigated whether the Pst or Tat system is functional in *E. chaffeensis*. As *E. chaffeensis* deletion mutants of these two systems are not available, we used *P. aeruginosa* as a surrogate system to study whether *E. chaffeensis* PstB and TatC are functional. In *P. aeruginosa*, TatC is critical for bacterial swimming motility and biofilm formation [21]. Inactivation of the Pst system leads to reduced motility and biofilm formation defects [9,34,35]. We then expressed PstB or TatC from *E. chaffeensis* or *P. aeruginosa* in a *pstB*::Tn mutant or a *tatC*::Tn mutant of the *P. aeruginosa* reference strain PA14, respectively [26]. We first examined whether *E. chaffeensis* PstB or TatC can restore the swimming motilities of the *P. aeruginosa pstB*::Tn or *tatC*::Tn mutant, respectively. The results showed that relative to the wild-type PA14 strain, the swimming motility of the *pstB*::Tn or *tatC*::Tn mutant was markedly impaired, which was restored upon heterologous expression of *E. chaffeensis* PstB or TatC, as well as homologous expression of *P. aeruginosa* PstB or TatC in the corresponding mutant strain (Figure 4a).

We then examined the ability of *E. chaffeensis* PstB or TatC to rescue biofilm formation of the *P. aeruginosa pstB*::Tn or *tatC*::Tn mutant. Relative to the wild-type PA14 strain, the biofilm formation of the *pstB*::Tn or *tatC*::Tn mutant was significantly impaired, which were restored upon heterologous expression of *E. chaffeensis* PstB or TatC, as well as homologous expression of *P. aeruginosa* PstB or TatC in the corresponding mutant strain (Figure 4b).

We also examined the ability of *E. chaffeensis* PstB or TatC to rescue survival under oxidative stress of the *P. aeruginosa pstB*::Tn or *tatC*::Tn mutant. The *P. aeruginosa* strains were grown to an OD_600_ of 1.0 and treated with 50 mM H_2_O_2_ at 37 °C for 30 min. Relative to the wild-type PA14 strain, the survival rate of the *pstB*::Tn or *tatC*::Tn mutant was markedly reduced, which was restored upon heterologous expression of *E. chaffeensis* PstB or TatC, as well as homologous expression of *P. aeruginosa* PstB or TatC in the corresponding mutant strain (Figure 4c). All these results confirm that *E. chaffeensis* PstB and TatC retain conserved biological activity.

### 3.5. PstB and TatC Are Essential for E. chaffeensis Intracellular Survival

**Figure 5:**
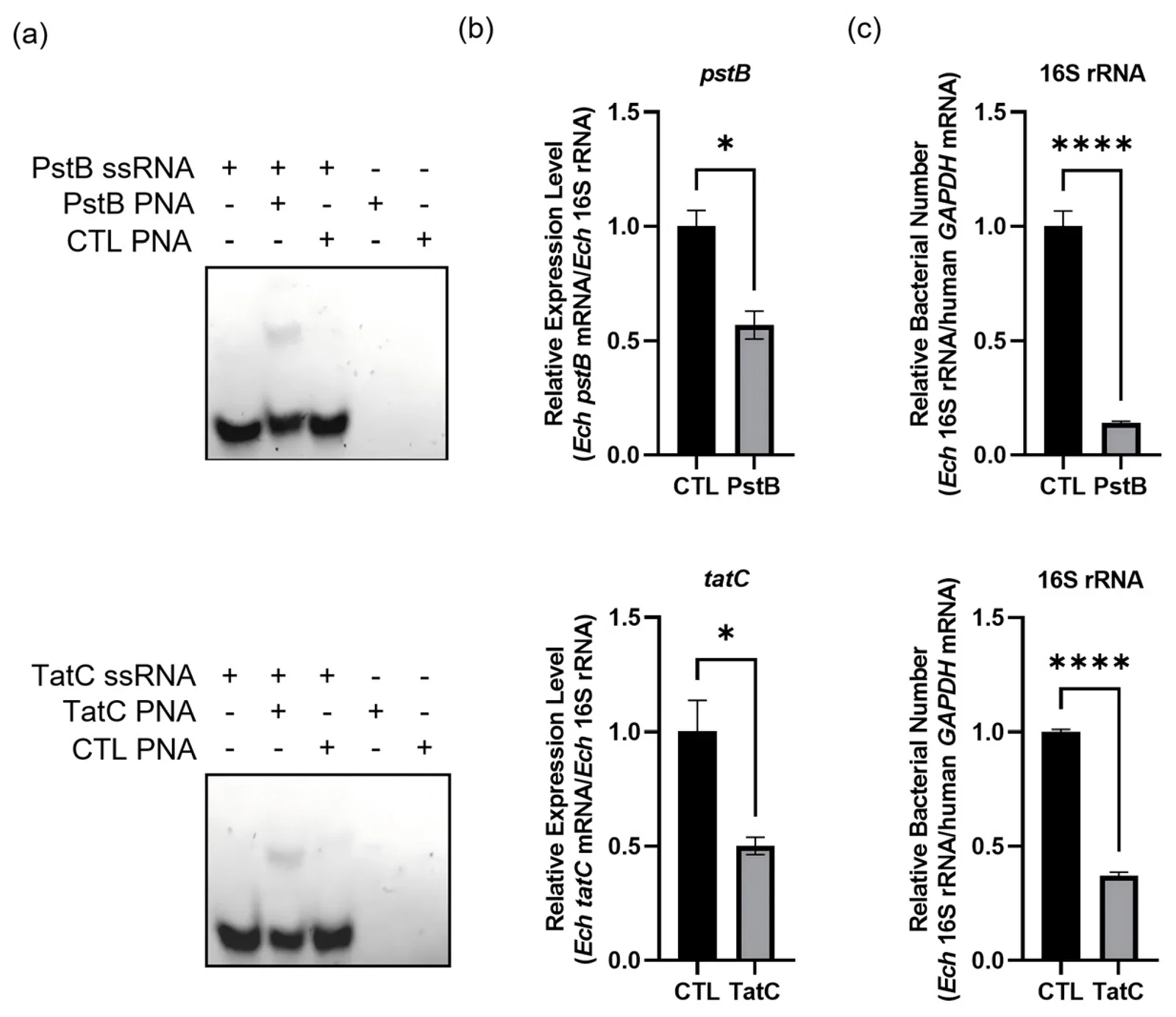
Knockdown of PstB or TatC reduces *E. chaffeensis* intracellular survival. (**a**) PstB PNA or TatC PNA binds to its corresponding ssRNA. ssRNA probe (40 pmol) alone, with its corresponding PNA (20 pmol) or with CTL PNA (20 pmol), was annealed from 95 °C to 35 °C. The same amount of corresponding PNA (20 pmol) or CTL PNA (20 pmol) alone was treated at the same condition. Shifted bands are indicated by arrowheads. (**b**) THP-1 cells synchronously infected with CTL PNA-, PstB PNA-, or TatC PNA-transfected *E. chaffeensis*. The mRNA expression levels of *pstB* or *tatC* were determined with qRT-PCR at 36 h p.i. and normalized against *E. chaffeensis* 16S rRNA levels. Relative values to the amount of CTL PNA-transfected *E. chaffeensis* are shown. (**c**) Relative bacterial number was determined as the amount of *E. chaffeensis* 16S rRNA normalized against that of human *GAPDH* mRNA. All data are presented as mean ± standard deviation (n = 3), with significant difference determined by Student’s *t*-test (* *p* < 0.05, **** *p* < 0.0001).

We then investigated the roles of the Pst or Tat system during *E. chaffeensis* intracellular survival. We designed PstB PNA and TatC PNA that specifically bind to the regions following the translation start codons of *pstB* and *tatC*, respectively (Supplementary Table S1). We confirmed the specificity by showing that PstB PNA bound to the PstB ssRNA (synthesized *pstB* mRNA fragment, 84–116 bp following the start codon) and that TatC PNA bound to the TatC ssRNA (synthesized *tatC* mRNA fragment, 216–247 bp following the start codon) (Figure 5a). Transfection of host cell-free *E. chaffeensis* with PstB PNA or TatC PNA reduced the mRNA levels of *pstB* or *tatC* to 56.9% or 49.9%, respectively, at 36 h p.i. (Figure 5b). We then investigated whether the reduction in the expression of *pstB* or *tatC* influenced bacterial intracellular survival. At 36 h p.i., the bacterial number of *E. chaffeensis* transfected with PstB PNA or TatC PNA was reduced to 14.1% or 37.1% compared with that of *E. chaffeensis* transfected with CTL PNA (Figure 5c), indicating that PstB and TatC are important for *E. chaffeensis* intracellular survival.

## 4. Discussion

As a global regulator, CtrA plays a critical role in the survival of *E. chaffeensis* and exhibits unique features distinct from its counterparts in other α-proteobacteria. In *C*. *crescentus*, CtrA mainly controls genes involved in the cell cycle, polar development, and cell division [5], with strict temporal and hierarchical regulation tightly coupled to the cell cycle, which is suited to its free-living lifestyle. Available data suggest that *E. chaffeensis* CtrA has incorporated multiple genes associated with intracellular survival, oxidative stress resistance, nutrient acquisition, and transport into its regulon [6,7,8]. The integration of these genes into the CtrA regulon indicates that *E. chaffeensis* employs its global regulator CtrA to coordinately harness multiple important physiological processes. This regulatory strategy enhances the efficiency of *E. chaffeensis* in responding to the intracellular environment, thereby conferring a critical adaptive advantage for its survival within host cells and subsequent infection. CckA functions as the specific histidine kinase paired with CtrA. In *C. crescentus*, CckA activity is coordinately modulated by c-di-GMP, the pseudokinase DivL, and the response regulator DivK [36]. However, the upstream signals sensed by *E. chaffeensis* CckA remain unknown and will be explored in future work.

This study supports a role for the Pst system in *E. chaffeensis* intracellular survival which is consistent with a model in which high-affinity Pi acquisition is important during infection. The Pst system is a bacteria-specific high-affinity ABC transporter that takes up Pi under low-phosphate conditions to sustain bacterial survival and proliferation [37,38]. In *C. crescentus*, the PhoU and Pst system jointly mediate stalk elongation under phosphate starvation to enhance nutrient uptake [39]. In uropathogenic *E. coli*, inactivation of Pst reduces type I fimbriae and attenuates bacterial virulence [40]. In *M*. *tuberculosis*, the Pst system has been targeted for vaccine development, with an mRNA vaccine MT.P495 targeting *M. tuberculosis* PstS1 showing promising potential [41]. In host cells, *E. chaffeensis* replicates within *E. chaffeensis*-containing vesicles (ECVs), which represent a low-phosphate environment [16]. Host cells recognize *E. chaffeensis* Ech_1067, a penicillin-binding protein, and then upregulate the phosphate transporter PIT1, which transports Pi from ECVs to the cytosol to inhibit bacterial growth [28]. In this context, the Pst system may help *E. chaffeensis* maintain Pi acquisition within the phosphate-limited ECVs.

This study suggests that the Tat system contributes substantially to *E. chaffeensis* intracellular survival. The functional role of the Tat system in mediating bacterial pathogenicity has been demonstrated in multiple bacteria [22,41]. In *Yersinia pseudotuberculosis*, the Tat system is functional, required for motility, and contributes to acid resistance [42]. In *A. phagocytophilum*, the Tat system components are expressed in the salivary glands of ticks during transmission feeding to mice, and are presumed to transport virulence determinants from the cytoplasm into the periplasm [24]. In *Legionella pneumophila*, the Tat system is essential for intracellular replication and biofilm formation [43]. *E. chaffeensis* is an obligate intracellular bacterium that relies heavily on host cells for nutrition [44], yet it retains a complete arginine biosynthesis pathway. No authentic Tat substrates have been experimentally identified in *E. chaffeensis*. Our previous ChIP-seq analysis identified multiple potential CtrA-regulated downstream genes whose products harbor typical Tat substrate recognition motifs, such as *petA*. PetA has been experimentally validated as an essential Tat substrate in diverse bacteria [45]. Tat inactivation leads to mislocalization of PetA and severe aerobic growth defects [46]. These proteins represent candidate Tat substrates that warrant experimental validation.

Due to the lack of effective genetic manipulation methods in *E. chaffeensis*, the functions of its proteins can only be studied using surrogate systems. Heterologous complementation in *P. aeruginosa* indicates that *E. chaffeensis* PstB and TatC retain conserved activity sufficient to restore phenotypes dependent on these systems. The reporter assay was performed in *E. coli* BL21 (DE3), while *E. chaffeensis* promoters may not function identically in *E. coli* and promoter activation may depend on additional *Ehrlichia*-specific factors. New approaches for studying the functions of *E. chaffeensis* proteins remain to be developed.

## 5. Conclusions

In this study, we found that CtrA regulates the expression of genes encoding components of the Pst and Tat systems in *E. chaffeensis*. Heterologous complementation assays suggest that *E. chaffeensis* PstB and TatC retain conserved functional activity, while PNA-mediated knockdown of *pstB* or *tatC* impaired bacterial intracellular survival. These findings expand our understanding of the noncanonical CtrA regulon in *E. chaffeensis* and suggest that CtrA-coordinated regulation of nutrient acquisition and protein translocation pathways contributes to adaptation within host cells.

## Data Availability

The data presented in the study are included in the article and Supplementary Materials; further inquiries can be directed to the corresponding author.

## Supplementary Materials

The following supporting information can be downloaded at: https://www.mdpi.com/article/10.3390/microorganisms14081664/s1.

## Abbreviations

The following abbreviations are used in this manuscript:

HME	human monocytic ehrlichiosis
Pst system	phosphate-specific transport system
Tat system	twin-arginine translocation system
ChIP-seq	chromatin immunoprecipitation coupled to deep sequencing
Pi	inorganic phosphate
PNA	peptide nucleic acid
FBS	fetal bovine serum
LB	Luria–Bertani
CTL-PNA	control PNA
cDNA	complementary DNA
qPCR	Quantitative PCR
IPTG	isopropyl-thio-β-D-galactoside
SDS-PAGE	SDS-polyacrylamide gel electrophoresis
ECVs	*E. chaffeensis*-containing vesicles

## References

[1] Ismail N., Bloch K.C., McBride J.W. (2010). Human ehrlichiosis and anaplasmosis. Clin. Lab. Med..

[2] Gygax L., Schudel S., Kositz C., Kuenzli E., Neumayr A. (2024). Human monocytotropic ehrlichiosis-A systematic review and analysis of the literature. PLoS Neglected Trop. Dis..

[3] Walker D.H., Dumler J.S. (1996). Emergence of the ehrlichioses as human health problems. Emerg. Infect. Dis..

[4] Farrera-Calderon R.G., Pallegar P., Westbye A.B., Wiesmann C., Lang A.S., Beatty J.T. (2021). The CckA-ChpT-CtrA Phosphorelay Controlling Rhodobacter capsulatus Gene Transfer Agent Production Is Bidirectional and Regulated by Cyclic di-GMP. J. Bacteriol..

[5] Laub M.T., Chen S.L., Shapiro L., McAdams H.H. (2002). Genes directly controlled by CtrA, a master regulator of the Caulobacter cell cycle. Proc. Natl. Acad. Sci. USA.

[6] Liang Q., Yan J., Zhang S., Yang N., Li M., Jin Y., Bai F., Wu W., Cheng Z. (2022). CtrA activates the expression of glutathione S-transferase conferring oxidative stress resistance to *Ehrlichia chaffeensis*. Front. Cell. Infect. Microbiol..

[7] Cheng Z., Miura K., Popov V.L., Kumagai Y., Rikihisa Y. (2011). Insights into the CtrA regulon in development of stress resistance in obligatory intracellular pathogen *Ehrlichia chaffeensis*. Mol. Microbiol..

[8] Yan J., Liang Q., Chai Z., Duan N., Li X., Liu Y., Yang N., Li M., Jin Y., Bai F. (2022). Glutathione Synthesis Regulated by CtrA Protects *Ehrlichia chaffeensis* from Host Cell Oxidative Stress. Front. Microbiol..

[9] Kritmetapak K., Kumar R. (2021). Phosphate as a Signaling Molecule. Calcif. Tissue Int..

[10] Gardner S.G., Miller J.B., Dean T., Robinson T., Erickson M., Ridge P.G., McCleary W.R. (2015). Genetic analysis, structural modeling, and direct coupling analysis suggest a mechanism for phosphate signaling in *Escherichia coli*. BMC Genet..

[11] Nikata T., Sakai Y., Shibat K., Kato J., Kuroda A., Ohtake H. (1996). Molecular analysis of the phosphate-specific transport (pst) operon of *Pseudomonas aeruginosa*. Mol. Gen. Genet..

[12] Runyen-Janecky L.J., Boyle A.M., Kizzee A., Liefer L., Payne S.M. (2005). Role of the Pst system in plaque formation by the intracellular pathogen Shigella flexneri. Infect. Immun..

[13] Lu J., Liu M., Wang Y., Pang Y., Zhao Z. (2014). Mechanisms of fluoroquinolone monoresistance in *Mycobacterium tuberculosis*. FEMS Microbiol. Lett..

[14] Yan Z., Yao X., Pan R., Zhang J., Ma X., Dong N., Wei J., Liu K., Qiu Y., Sealey K. (2023). Subunit Vaccine Targeting Phosphate ABC Transporter ATP-Binding Protein, PstB, Provides Cross-Protection against *Streptococcus suis* Serotype 2, 7, and 9 in Mice. Vet. Sci..

[15] Cheng Z., Kumagai Y., Lin M., Zhang C., Rikihisa Y. (2006). Intra-leukocyte expression of two-component systems in *Ehrlichia chaffeensis* and *Anaplasma phagocytophilum* and effects of the histidine kinase inhibitor closantel. Cell. Microbiol..

[16] Roder J., Felgner P., Hensel M. (2021). Single-cell analyses reveal phosphate availability as critical factor for nutrition of *Salmonella enterica* within mammalian host cells. Cell. Microbiol..

[17] Frain K.M., Robinson C., van Dijl J.M. (2019). Transport of Folded Proteins by the Tat System. Protein J..

[18] Sargent F., Stanley N.R., Berks B.C., Palmer T. (1999). Sec-independent protein translocation in *Escherichia coli*. A distinct and pivotal role for the TatB protein. J. Biol. Chem..

[19] Blaudeck N., Kreutzenbeck P., Muller M., Sprenger G.A., Freudl R. (2005). Isolation and characterization of bifunctional *Escherichia coli* TatA mutant proteins that allow efficient tat-dependent protein translocation in the absence of TatB. J. Biol. Chem..

[20] Ma Q., Zhai Y., Schneider J.C., Ramseier T.M., Saier M.H. (2003). Protein secretion systems of *Pseudomonas aeruginosa* and *P. fluorescens*. Biochim. Biophys. Acta.

[21] Ochsner U.A., Snyder A., Vasil A.I., Vasil M.L. (2002). Effects of the twin-arginine translocase on secretion of virulence factors, stress response, and pathogenesis. Proc. Natl. Acad. Sci. USA.

[22] Yan X., Hu S., Yang Y., Xu D., Li H., Liu W., He X., Li G., Cai W., Bu Z. (2020). The Twin-Arginine Translocation System Is Important for Stress Resistance and Virulence of Brucella melitensis. Infect. Immun..

[23] Helminiak L., Mishra S., Kim H.K. (2022). Pathogenicity and virulence of Rickettsia. Virulence.

[24] Mastronunzio J.E., Kurscheid S., Fikrig E. (2012). Postgenomic analyses reveal development of infectious *Anaplasma phagocytophilum* during transmission from ticks to mice. J. Bacteriol..

[25] Teymournejad O., Rikihisa Y. (2020). *Ehrlichia chaffeensis* Uses an Invasin To Suppress Reactive Oxygen Species Generation by Macrophages via CD147-Dependent Inhibition of Vav1 To Block Rac1 Activation. mBio.

[26] Liberati N.T., Urbach J.M., Miyata S., Lee D.G., Drenkard E., Wu G., Villanueva J., Wei T., Ausubel F.M. (2006). An ordered, nonredundant library of *Pseudomonas aeruginosa* strain PA14 transposon insertion mutants. Proc. Natl. Acad. Sci. USA.

[27] Weng Y., Chen F., Liu Y., Zhao Q., Chen R., Pan X., Liu C., Cheng Z., Jin S., Jin Y. (2016). *Pseudomonas aeruginosa* Enolase Influences Bacterial Tolerance to Oxidative Stresses and Virulence. Front. Microbiol..

[28] Li M., Yang N., Li X., Duan N., Qin S., Wang M., Zhou Y., Jin Y., Wu W., Jin S. (2024). Host Cells Upregulate Phosphate Transporter PIT1 to Inhibit *Ehrlichia chaffeensis* Intracellular Growth. Int. J. Mol. Sci..

[29] Duan N., Ma X., Cui H., Wang Z., Chai Z., Yan J., Li X., Feng Y., Cao Y., Jin Y. (2021). Insights into the mechanism regulating the differential expression of the P28-OMP outer membrane proteins in obligatory intracellular pathogen *Ehrlichia chaffeensis*. Emerg. Microbes Infect..

[30] Zhang Y., Zhang C., Du X., Zhou Y., Kong W., Lau G.W., Chen G., Kohli G.S., Yang L., Wang T. (2019). Glutathione Activates Type III Secretion System Through Vfr in *Pseudomonas aeruginosa*. Front. Cell. Infect. Microbiol..

[31] McClure E.E., Chavez A.S.O., Shaw D.K., Carlyon J.A., Ganta R.R., Noh S.M., Wood D.O., Bavoil P.M., Brayton K.A., Martinez J.J. (2017). Engineering of obligate intracellular bacteria: Progress, challenges and paradigms. Nat. Rev. Microbiol..

[32] Pelc R.S., McClure J.C., Kaur S.J., Sears K.T., Rahman M.S., Ceraul S.M. (2015). Disrupting protein expression with Peptide Nucleic Acids reduces infection by obligate intracellular Rickettsia. PLoS ONE.

[33] Sharma P., Teymournejad O., Rikihisa Y. (2017). Peptide Nucleic Acid Knockdown and Intra-host Cell Complementation of Ehrlichia Type IV Secretion System Effector. Front. Cell. Infect. Microbiol..

[34] Haddad A., Jensen V., Becker T., Haussler S. (2009). The Pho regulon influences biofilm formation and type three secretion in *Pseudomonas aeruginosa*. Environ. Microbiol. Rep..

[35] Peng Y.C., Lu C., Li G., Eichenbaum Z., Lu C.D. (2017). Induction of the pho regulon and polyphosphate synthesis against spermine stress in *Pseudomonas aeruginosa*. Mol. Microbiol..

[36] Pierce D.L., O’Donnol D.S., Allen R.C., Javens J.W., Quardokus E.M., Brun Y.V. (2006). Mutations in DivL and CckA rescue a divJ null mutant of *Caulobacter crescentus* by reducing the activity of CtrA. J. Bacteriol..

[37] Yuan Z.C., Zaheer R., Finan T.M. (2006). Regulation and properties of PstSCAB, a high-affinity, high-velocity phosphate transport system of *Sinorhizobium meliloti*. J. Bacteriol..

[38] Healy C.M., Pham E.A., Dye K.J., Rouchon C.N., McMillan B., Frank K.L. (2025). The adjacent ATP-binding protein-encoding genes of the *Enterococcus faecalis* phosphate-specific transport (pst) locus have non-overlapping cellular functions. J. Bacteriol..

[39] Gonin M., Quardokus E.M., O’Donnol D., Maddock J., Brun Y.V. (2000). Regulation of stalk elongation by phosphate in *Caulobacter crescentus*. J. Bacteriol..

[40] Crepin S., Porcheron G., Houle S., Harel J., Dozois C.M. (2017). Altered Regulation of the Diguanylate Cyclase YaiC Reduces Production of Type 1 Fimbriae in a Pst Mutant of Uropathogenic *Escherichia coli* CFT073. J. Bacteriol..

[41] Shahrear S., Islam A. (2023). Modeling of MT. P495, an mRNA-based vaccine against the phosphate-binding protein PstS1 of *Mycobacterium tuberculosis*. Mol. Divers..

[42] Lavander M., Ericsson S.K., Broms J.E., Forsberg A. (2006). The twin arginine translocation system is essential for virulence of *Yersinia pseudotuberculosis*. Infect. Immun..

[43] De Buck E., Maes L., Meyen E., Van Mellaert L., Geukens N., Anne J., Lammertyn E. (2005). *Legionella pneumophila* Philadelphia-1 tatB and tatC affect intracellular replication and biofilm formation. Biochem. Biophys. Res. Commun..

[44] Rikihisa Y. (2021). The “Biological Weapons” of *Ehrlichia chaffeensis*: Novel Molecules and Mechanisms to Subjugate Host Cells. Front. Cell. Infect. Microbiol..

[45] De Buck E., Vranckx L., Meyen E., Maes L., Vandersmissen L., Anne J., Lammertyn E. (2007). The twin-arginine translocation pathway is necessary for correct membrane insertion of the Rieske Fe/S protein in *Legionella pneumophila*. FEBS Lett..

[46] Luo Q., Dong Y., Chen H., Gao H. (2013). Mislocalization of Rieske protein PetA predominantly accounts for the aerobic growth defect of Tat mutants in *Shewanella oneidensis*. PLoS ONE.

