# Supplementary material for: Pst and Tat Systems Are Essential for Ehrlichia chaffeensis Intracellular Survival and Regulated by CtrA
Source: Microorganisms. 2026 Jul 30;14(8):1664. doi: 10.3390/microorganisms14081664 (PMC13515596; doi:10.3390/microorganisms14081664)
Supplement: Supplementary file 1 [file microorganisms-14-01664-s001.zip › microorganisms-4378466-supplementary.pdf]

Supplementary Figures

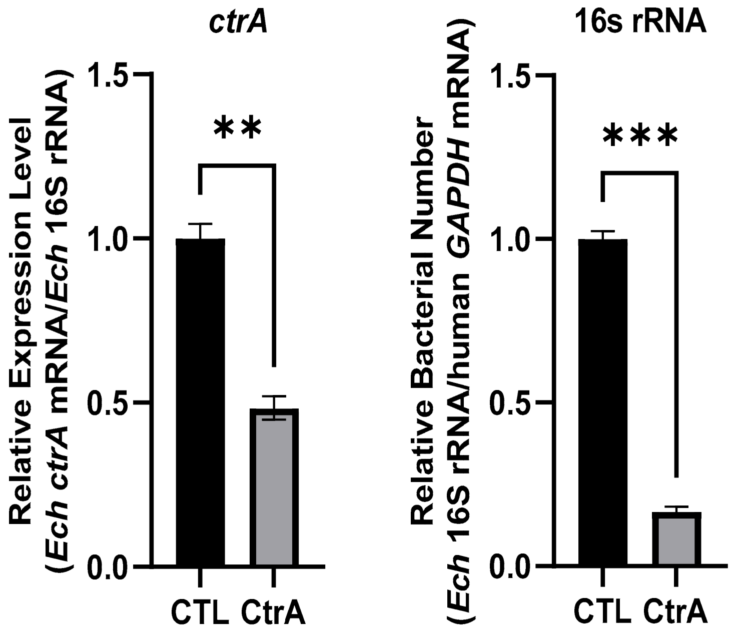

Figure S1. Transfection with CtrA−PNA inhibits the expression of *ctrA* and reduced *E. chaffeensis* intracellular survival. THP-1 cells synchronously infected with CTL-PNA or CtrA-PNA transfected *E. chaffeensis*. The mRNA expression level of *ctrA* was determined with qRT-PCR at 36 h p.i. and normalized against the *E. chaffeensis 16S* rRNA level(left). Relative values to the amount of CTL-PNA transfected *E. chaffeensis* are shown. Relative bacterial number was determined as the amount of *E. chaffeensis* 16S rRNA normalized against that of human *GAPDH* mRNA(right). All data are presented as mean ± SD (n = 3), with significant difference determined by Student's *t*-test (**$p < 0.01$, ***$p < 0.001$).

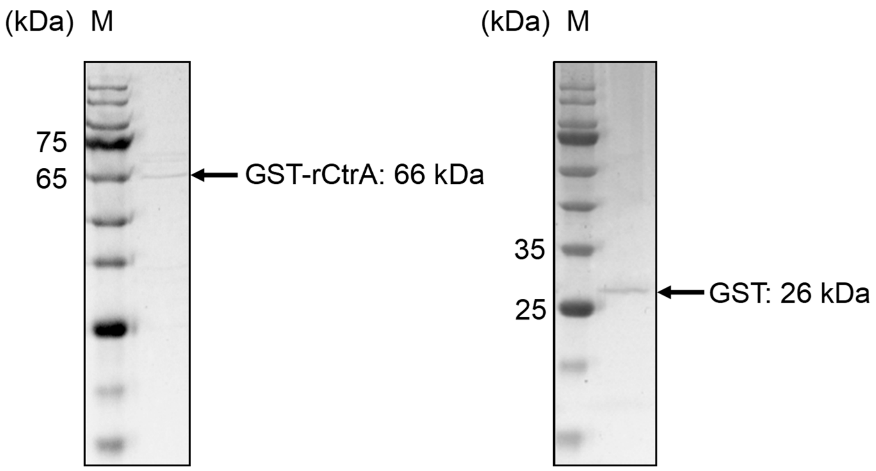

Figure S2. Purification of recombinant *E.chaffeensis* GST-rCtrA and GST. *E.chaffeensis* GST-rCtrA and GST gene were cloned into the pET41a(+) vector, expressed, and purified using Ni-affinity chromatography. The purified proteins were subjected to SDS-PAGE, followed by coomassie blue staining. M stands for protein marker. The purified proteins are indicated by arrows.

Table S1. Bacterial strains, plasmids, PNAs and ssRNAs used in this study.

Strains/Plasmids/PNAs /ssRNAs	Description	Source (Reference)
E. coli strains		
BL21(DE3)/GST	BL21(DE3) expressing GST; Kan^r	[6]
BL21(DE3)/rCtrA	BL21(DE3) expressing rCtrA; Kan^r	[6]
BL21(DE3)/ pACYCDuet-1	BL21(DE3) expressing; Chl^r	[6]
BL21(DE3)/ pACYCDuet-1-rCtrA	BL21(DE3) expressing rCtrA; Chl^r	[6]
DH5α/pQE60-*pstA* promoter-EGFP	DH5α / pQE60-EGFP containing *pstA* promoter; Amp^r	This study
DH5α/pQE60-*pstB* promoter-EGFP	DH5α / pQE60-EGFP containing *pstB* promoter; Amp^r	This study
DH5α/pQE60-*pstC* promoter-EGFP	DH5α / pQE60-EGFP containing *pstC* promoter; Amp^r	This study
DH5α/pQE60-*tatA* promoter-EGFP	DH5α / pQE60-EGFP containing *tatA* promoter; Amp^r	This study
DH5α/pQE60-*tatB* promoter-EGFP	DH5α / pQE60-EGFP containing *tatB* promoter; Amp^r	This study
DH5α/pQE60-*tatC* promoter-EGFP	DH5α / pQE60-EGFP containing *tatC* promoter; Amp^r	This study
DH5α/pQE60-*p28* promoter-EGFP	DH5α / pQE60-EGFP containing *p28* promoter; Amp^r	[6]
BL21(DE3)/ pACYCDuet-1/ *pstA*	BL21(DE3) harboring pACYCDuet-1 and pQE60-*pstA* promoter-EGFP plasmids; Chl^r, Amp^r	This study
BL21(DE3)/ pACYCDuet-1/ *pstB*	BL21(DE3) harboring pACYCDuet-1 and pQE60-*pstB* promoter-EGFP plasmids; Chl^r, Amp^r	This study
BL21(DE3)/ pACYCDuet-1/ *pstC*	BL21(DE3) harboring pACYCDuet-1 and pQE60-*pstC* promoter-EGFP plasmids; Chl^r, Amp^r	This study
BL21(DE3)/ pACYCDuet-1/ *tatA*	BL21(DE3) harboring pACYCDuet-1 and pQE60-*tatA* promoter-EGFP plasmids; Chl^r, Amp^r	This study
BL21(DE3)/ pACYCDuet-1/ *tatB*	BL21(DE3) harboring pACYCDuet-1 and pQE60-*tatB* promoter-EGFP plasmids; Chl^r, Amp^r	This study
BL21(DE3)/ pACYCDuet-1/ *tatC*	BL21(DE3) harboring pACYCDuet-1 and pQE60-*tatC* promoter-EGFP plasmids; Chl^r, Amp^r	This study
BL21(DE3)/ pACYCDuet-1/ *p28*	BL21(DE3) harboring pACYCDuet-1 and pQE60-*p28* promoter-EGFP plasmids; Chl^r, Amp^r	[6]
BL21(DE3)/ pACYCDuet-1-rCtrA / *pstA*	BL21(DE3) harboring pACYCDuet-1-rCtrA and pQE60-*pstA* promoter-EGFP plasmids; Chl^r, Amp^r	This study
BL21(DE3)/ pACYCDuet-1-rCtrA / *pstB*	BL21(DE3) harboring pACYCDuet-1-rCtrA and pQE60-*pstB* promoter-EGFP plasmids; Chl^r, Amp^r	This study
BL21(DE3)/ pACYCDuet-1-rCtrA /*pstC*	BL21(DE3) harboring pACYCDuet-1-rCtrA and pQE60-*pstC* promoter-EGFP plasmids; Chl^r , Amp^r	This study
BL21(DE3)/ pACYCDuet-1-rCtrA /*tatA*	BL21(DE3) harboring pACYCDuet-1-rCtrA and pQE60-*tatA* promoter-EGFP plasmids; Chl^r, Amp^r	This study
BL21(DE3)/ pACYCDuet-1-rCtrA /*tatB*	BL21(DE3) harboring pACYCDuet-1-rCtrA and pQE60-*tatB* promoter-EGFP plasmids; Chl^r, Amp^r	This study
BL21(DE3)/ pACYCDuet-1-rCtrA /*tatC*	BL21(DE3) harboring pACYCDuet-1-rCtrA and pQE60-*tatC* promoter-EGFP plasmids; Chl^r, Amp^r	This study
BL21(DE3)/ pACYCDuet-1-rCtrA /*p28*	BL21(DE3) harboring pACYCDuet-1-rCtrA and pQE60-*p28* promoter-EGFP plasmids; Chl^r, Amp^r	[6]
P. aeruginosa strains		
PA14/pUCP20	Wild type *P. aeruginosa* strain harboring pUCP20	Lab stock
PA14 *pstB*::Tn/ pUCP20	PA14 with *pstB* disrupted by insertion of Tn harboring pUCP20	This study
PA14 *tatC*::Tn/pUCP20	PA14 with *tatC* disrupted by insertion of Tn harboring pUCP20	This study
PA14 *pstB*::Tn/ pUCP20-$pstB_{Ech}$	PA14 with *pstB* disrupted by insertion of Tn harboring pUCP20- *E. chaffeensis pstB*	This study

PA14 *tatC*::Tn/ pUCP20-*tatC*$_{Ech}$	PA14 with *tatC* disrupted by insertion of Tn harboring pUCP20- *E. chaffeensis tatC*	This study
PA14 *pstB*::Tn/ pUCP20-*pstB*$_{Pa}$	PA14 with *pstB* disrupted by insertion of Tn harboring pUCP20-*P. aeruginosa* PA14 *pstB*	This study
PA14 *tatC*::Tn/ pUCP20-*tatA*$_{Pa}$	PA14 with *tatC* disrupted by insertion of Tn harboring pUCP20-*P. aeruginosa* PA14 *tatC*	This study
Plasmids		
pET-41a (+)	Clone vector; Kan^r	Novagen
pCtrA	pET-41a(+) harboring *ctrA* gene; Kan^r	This study
pACYCDuet-1	Clone vector; Chl^r	Novagen
pACYCDuet-1-rCtrA	pACYCDuet-1 harboring *ctrA* gene; Chl^r	This study
pQE60-EGFP	Amp^r	Lab stock
pQE60-*pstA* promoter-EGFP	pQE60-EGFP harboring *pstA* promoter; Amp^r	This study
pQE60-*pstB* promoter-EGFP	pQE60-EGFP harboring *pstB* promoter; Amp^r	This study
pQE60-*pstC* promoter-EGFP	pQE60-EGFP harboring *pstC* promoter; Amp^r	This study
pQE60-*tatA* promoter-EGFP	pQE60-EGFP harboring *tatA* promoter; Amp^r	This study
pQE60-*tatB* promoter-EGFP	pQE60-EGFP harboring *tatB* promoter; Amp^r	This study
pQE60-*tatC* promoter-EGFP	pQE60-EGFP harboring *tatC* promoter; Amp^r	This study
pQE60-*p28* promoter-EGFP	pQE60-EGFP harboring *p28* promoter; Amp^r	This study
PNA	Sequence (5' →3')	Function
CtrA	CTGCCTTTGCACATGC	CtrA knockdown
Control (CTL)	GGCTCTATACAC	Negative control
PstB	GCTGTAACTTCTCTTT	PstB knockdown
TatC	ATAGTAGCAACTTTTA	TatC knockdown
ssRNA	Sequence (5' →3')	
PstB	GUAUAUAAAAGAGAAGUUACAGCUUUUAUAGG	Hybridization assay
TatC	GUAUAUUUAAAAGUUGCUACUAUGGCUGCUUU	Hybridization assay

Kan^r, kanamycin resistance; Amp^r, ampicillin resistance. Chl^r, chloramphenicol resistance.

The complementary bases to corresponding PNA are indicated by red letters.

Table S2. Primers used in this study.

Primers	Sequence (5'→3')	Function
pstA-promoter-F	AATGTTAACCTTTACTGTATATAG	EMSA
pstA-promoter-R	TAAACACAACTAAAAAGTCCAAC	EMSA
pstB-promoter-F	AGGGAAGAAATGTTCCTTTCC	EMSA
pstB-promoter-R	ATTAACCCTAATTAAATAACTACT	EMSA
pstC-promoter-F	AAATGATTTTCTAAGATTGCTAAC	EMSA
pstC-promoter-R	AGTTCACAAATTTTTTATATGTATAG	EMSA
tatA-promoter-F	TTAAGTCAAGCAAGATAAATATAG	EMSA
tatA-promoter-R	TGAGATATATTATAGTCCCAAAG	EMSA
tatB-promoter-F	AGCAGTAAAATTGGATAAAGTAC	EMSA
tatB-promoter-R	TTGTTTTAATTAATAAACATATCAAG	EMSA
tatC-promoter-F	ATGGTCCAGGTGAAAGTAAAC	EMSA
tatC-promoter-R	TTATAGATTACCTTTTTTACTATTG	EMSA
pstB-in-promoter-F	TGATGACATAGGTGTTTATTCTG	EMSA
pstB-in-promoter-R	ACATGGTTCATCCATAAGTAAC	EMSA
pQE60-pstA promoter-F	CCGCTCGAGTAAACACAACTAAAAAGTCCAAC	reporter assay

pQE60-pstA promoter-R	CCG<u>GAATTC</u>GTTATTATAAATGTTAACCTTTACT	reporter assay
PQE60-*pstB* promoter-F	CCG<u>CTCGAG</u>AGGGAAGAAATGTTCCTTTCC	reporter assay
pQE60-*pstB* promoter-R	CCG<u>GAATTC</u>ATTAACCCTAATTAAATAACTACTC	reporter assay
pQE60-*pstC* promoter-F	CCG<u>CTCGAG</u>AATTGTACCATACCAGCATTAG	reporter assay
pQE60-*pstC* promoter-R	CCG<u>GAATTC</u>GGTATTTATAAATAAAGTTCACAAATTTT	reporter assay
pQE60-*tatA* promoter-F	CCG<u>CTCGAG</u>ATTCTTGTATAGAATTACCGATAG	reporter assay
pQE60-*tatA* promoter-R	CCG<u>GAATTC</u>AATAGAACTCCTATAAAATATAAAAC	reporter assay
pQE60-tatB promoter-F	CCG<u>CTCGAG</u>AGCAGTAAAATTGGATAAAGTAC	reporter assay
pQE60-tatB promoter-R	GAA<u>TTCTTG</u>GAATTGTTTTAATTAATAAACAT	reporter assay
pQE60-*tatC* promoter-F	CCG<u>CTCGAG</u>ATGGTCCAGGTGAAAGTAAAC	reporter assay
pQE60-*tatC* promoter-R	CCG<u>GAATTC</u>TTATAGATTACCTTTTTTACTATTG	reporter assay
ctrA-qRT-F	TGCATGTGCAAAGGCAGTAG	qRT-PCR
ctrA-qRT-R	ACATCATAGTCATCATTCTTCG	qRT-PCR
16S rRNA-qRT-F	GGTGAGTAATGCGTAGGAATC	qRT-PCR
16S rRNA-qRT-R	GCTCATCTAATAGCGATAAATC	qRT-PCR
GAPDH-qRT-F	TCAAGGCTGAGAACGGGAAG	qRT-PCR
GAPDH-qRT-R	TGGACTCCACGACGTACTCA	qRT-PCR
pstA-qRT-F	TGTTGTTCTATAGTATCATTATTG	qRT-PCR
pstA-qRT-R	TATCAATGACTGCACTTGTTAG	qRT-PCR
pstB-qRT-F	AGAAAAAAGCTTAACAAGTGTTG	qRT-PCR
pstB-qRT-R	TGCAATAGCACGCGCTATAC	qRT-PCR
pstC-qRT-F	ATTCGGAATATTACCACTACTC	qRT-PCR
pstC-qRT-R	TTTCAAGTGTTGTATTAGCTATG	qRT-PCR
tatA-qRT-F	TGGTTCGTCTTCCGTTGATG	qRT-PCR
tatA-qRT-R	TAGTATTGTTTGGTGCAGGCA	qRT-PCR
tatB-qRT-F	ATTGTCATAATCCTAGTAGTATG	qRT-PCR
tatB-qRT-R	TTCTATGCAGTCCATGTAAAAC	qRT-PCR
tatC-qRT-F	TTTTAACATTAATGTCTAGGATTG	qRT-PCR
tatC-qRT-R	TTTGGCTTAACACATCAGGTG	qRT-PCR
pstB-Pa-F	CCC<u>AAGCTT</u>CTAGCCGTAGCGGCCGG	construct vector
pstB-Pa-R	CGG<u>AATTCC</u>TGGAAAACTGATTGAGGGAGCTTCC	construct vector
tatC-Pa-F	CGG<u>AATTCC</u>CGAGACGCCGCGTAATCC	construct vector
tatC-Pa-R	CCC<u>AAGCTT</u>TCACTGGCGGGTGGCC	construct vector
pstB-Ech-F	CGG<u>GTACCT</u>GCTGTAGAGTAGTTATTTAATTAG	construct vector
pstB-Ech-R	CGG<u>GATCCC</u>TATAACTGATCTACTATATACCT	construct vector
tatC-Ech-F	CGG<u>GTACCA</u>GTAAAAAAGGTAATCTATAATTATG	construct vector
tatC-Ech-R	CGG<u>GATCCT</u>CATTTAGAACGTTCAATATATTTA	construct vector

The enzymes sites are indicated by the underline.
